# Canine faecal contamination and parasitic risk in the city of Naples (southern Italy)

**DOI:** 10.1186/1746-6148-2-29

**Published:** 2006-09-22

**Authors:** Laura Rinaldi, Annibale Biggeri, Sabrina Carbone, Vincenzo Musella, Dolores Catelan, Vincenzo Veneziano, Giuseppe Cringoli

**Affiliations:** 1 Dipartimento di Patologia e Sanità Animale, Università degli Studi di Napoli "Federico II" – *CREMOPAR *Regione Campania, Napoli, Italy; 2 Dipartimento di Statistica "G. Parenti", Università di Firenze, Firenze, Italy

## Abstract

**Background:**

Dogs are associated with more than 60 zoonotic diseases among which, parasitosis and, in particular, helminthosis, can pose serious public-health concerns worldwide. Many canine gastrointestinal parasites eliminate their dispersion elements (eggs, larvae, oocysts) by the faecal route. The quantity of canine faeces deposited on public and private property in cities worldwide is both a perennial nuisance and an important health issue. Public sites such as playgrounds, parks, gardens, public squares and sandpits may be an important source of human infection. The aim of this study was to evaluate the extent of both canine faecal contamination in the city of Naples (southern Italy), and presence of canine parasitic elements, with particular regard to those which are potential agents of zoonosis. A regular grid of sub-areas (1 km × 700 m) was overlaid on the city map using a Geographical Information System (GIS). In each sub-area the straightest 1 km transect was drawn and digitalized on-screen in the GIS. Between February and May 2005 canine faeces were counted along the 1 km transects in 143 sub-areas, and 415 canine faecal samples were collected and submitted to coprological examinations. Negative binomial regression models and Gaussian random effects models were used to analyze the association between faeces count and human population density taking into account for extraPoisson variability. Logistic regression model was used to evaluate the association between positivity to parasitic elements and number of canine faeces.

**Results:**

Out of the 143 studied sub-areas, 141 (98.6%) contained canine faeces. There was a strong spatial gradient with 48% of the total variability accounted by between neighbourhood variability; a positive association between the number of faeces and the human population density was found. Seventy (over 415, 16.9%) canine faecal samples were positive for parasitic elements. There was no association between positivity to parasitic elements and the number of canine faeces. Eggs of *Toxocara canis*, *Toxascaris leonina*, *Ancylostoma caninum *and *Trichuris vulpis *were found, as well as oocysts of *Isospora canis*.

**Conclusion:**

In conclusion, the results of the present study, conducted using GIS both for planning and sampling and for evaluation and presentation of findings, showed the presence of canine faecal contamination in the city of Naples, and the presence of canine parasitic elements, some of which are potential agents of zoonosis.

## Background

Public-health problems caused by the impact of dogs on humans are both direct and indirect, e.g. environmental pollution, contact injuries, and zoonosis [1]. Dogs are associated with more than 60 zoonotic diseases [2] among which, parasitosis and, in particular, helminthosis, can pose serious public-health concerns worldwide [3-5]. Many canine gastrointestinal parasites eliminate their dispersion elements (eggs, larvae, oocysts) by the faecal route. The quantity of canine faeces deposited on public and private property in cities worldwide is both, a perennial nuisance and an important health issue [6]. Public sites such as playgrounds, parks, gardens, public squares, sandpits may be an important source of human infection [5,7,8].

Our aim was to evaluate the extent of both, canine faecal contamination in the city of Naples (southern Italy), and the consequent presence of canine parasitic elements (determined by coprological examinations), with particular regard to those which are potential agents of zoonosis. A Geographical Information System (GIS) was used in order to plan the study and to display the results [9].

## Results

**Figure 1:**
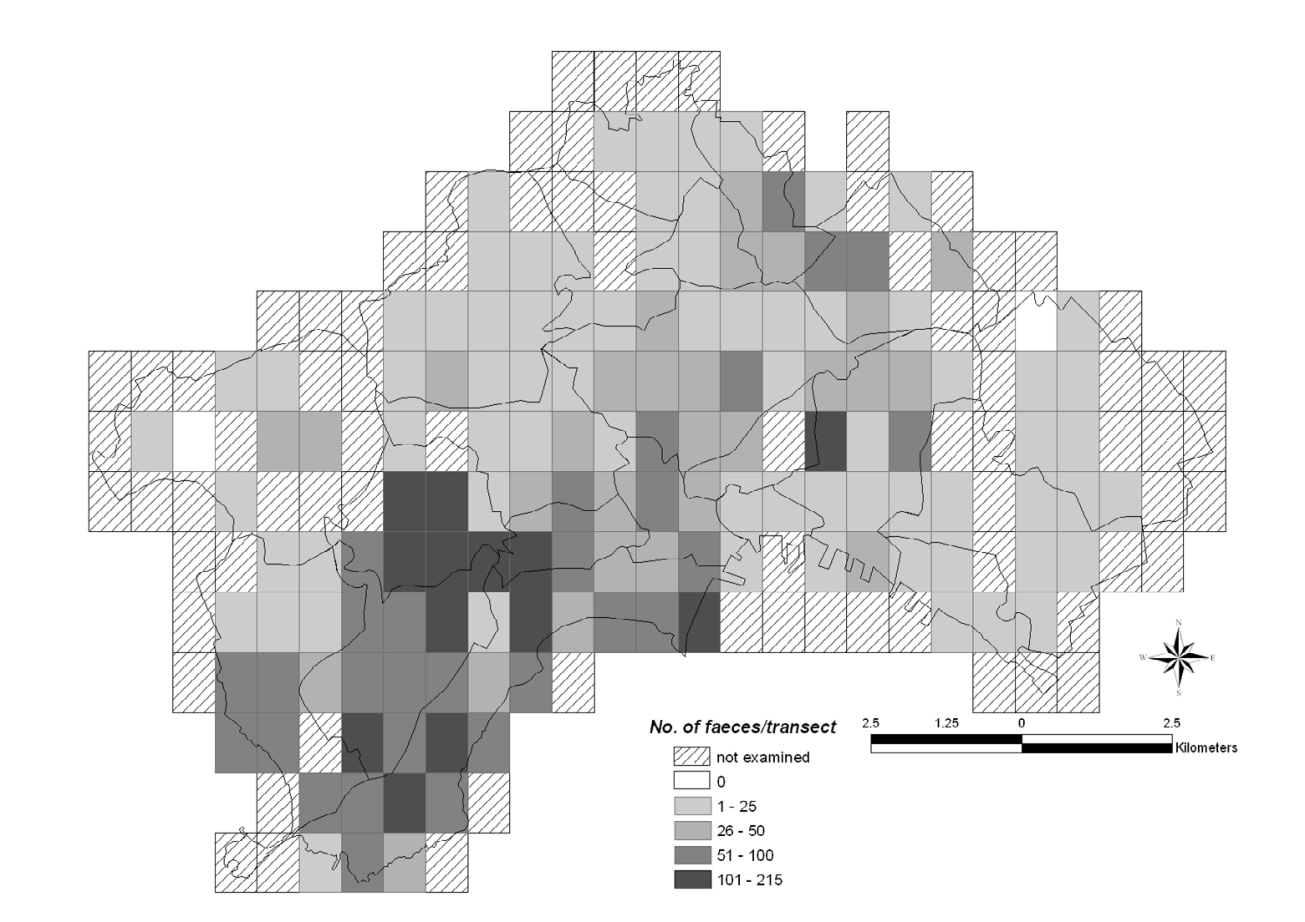
Occurrence of canine faecalization in the city of Naples, Italy, February–May, 2005. Faeces counts on regular grid of 1 km × 700 m (faeces/transect).

Out of the 143 studied sub-areas, 141 (98.6%, 95% CI = 94.5%, 99.8%) contained canine faeces. The total number of canine faeces counted was 5,861 (Median = 25.0; Minimum = 0, Maximum = 215). Fig. 1 is a choroplethic map showing the total number of faeces counted along each 1 km transect of each sub-area.

The results by both the negative binomial and the Gaussian linear random effects models were highly consistent, showing a significant contribution of the between neighbourhoods variability (the likelihood ratio test being: negative binomial model chi-square 57.97 and Gaussian linear random effect model chi-square 52.47; both with P < 0.001; since the test is on the boundary-value the P value was evaluated on a mixture of chi-square distributions with zero and 1 degree of freedom, see [10]). The percentage of variability accounted by neighbourhoods was estimated around 48% (95% CI = 30%, 68%) by the Gaussian linear random effect model. The results of the negative binomial regression model showed a positive association between the number of canine faeces and the human population density (likelihood ratio chi-square = 11.8; 1 df; P < 0.001). No improvement in goodness-of-fit was observed by using either male or female human population density instead of the total.

Out of the 143 sub-areas, 58 (40.6%, 95% CI = 32.5%, 49.1%) were positive for parasitic elements. The estimated association between positivity to parasite elements and the number of canine faeces in the neighbourhoods was not statistically significant (P = 0.138).

**Figure 2:**
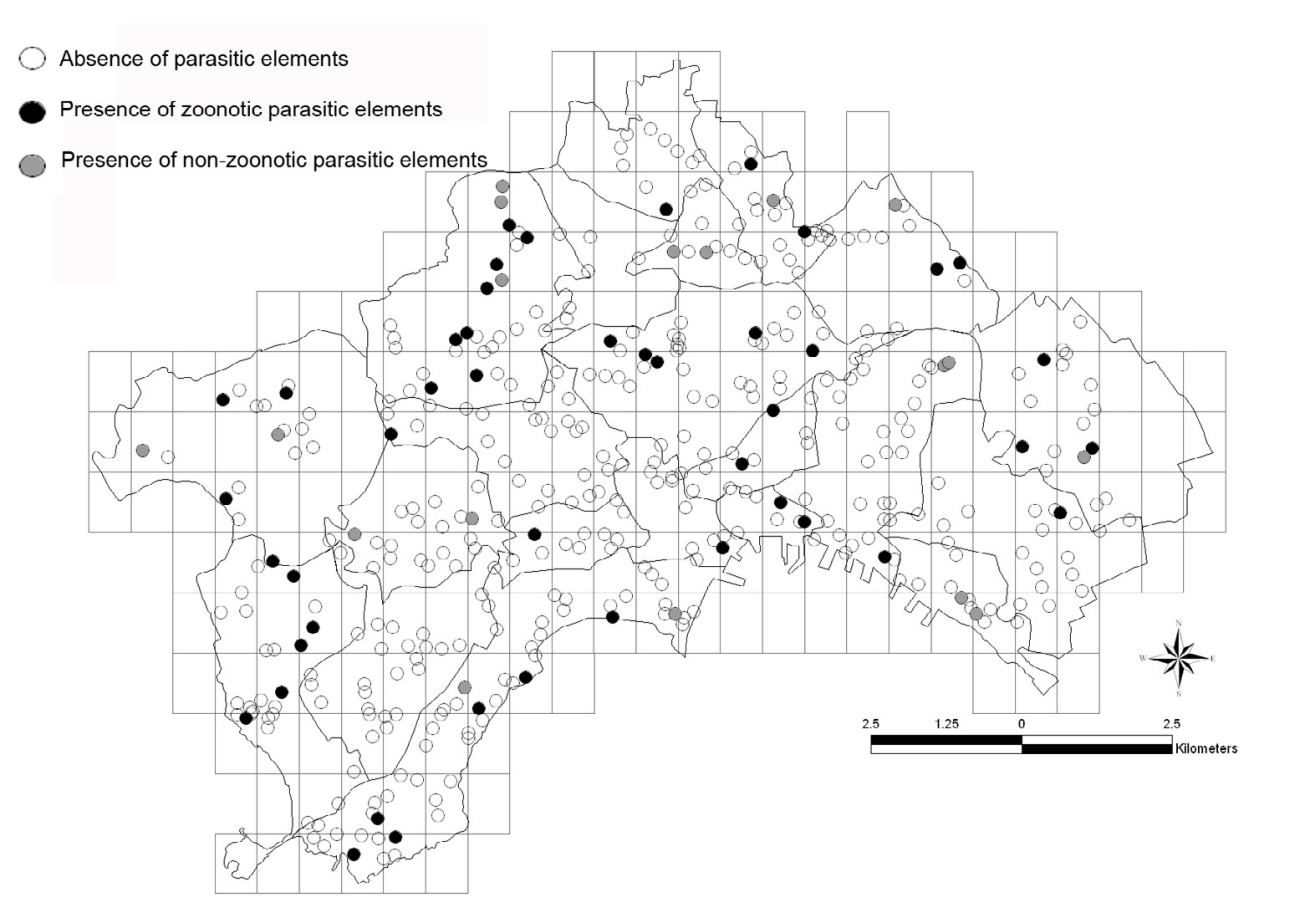
**Occurrence of canine faecalization in the city of Naples, Italy, February–May, 2005**. Location of negative (white) and positive (black and grey) samples for parasitic elements.

Fig. 2 is a point map showing the locations of all the collected faeces. Seventy (16.9%, 95% CI = 13.5%, 20.9%) samples among the total (415) were positive for parasitic elements. Eggs of zoonotic parasites (i.e. *Toxocara canis*, *Ancylostoma caninum *and *Trichuris vulpis*) were found, as well as parasitic elements of non-zoonotic parasites (i.e. eggs of *Toxascaris leonina *and oocysts of *Isospora canis*).

Table 1 shows, for each parasitic species, the number, percentage and 95% CI of positive sub-areas, the number, percentage and 95% CI of positive faecal samples, and the EPG/OPGs (25^th^, 50^th^, and 75^th ^percentiles).

The most frequent parasite species found in the examined faecal samples were *T. vulpis *(10.1%), followed by *I. canis *(4.1%), *A. caninum *(2.4%), *T. leonina *(1.4%) and *T. canis *(0.7%). Other zoonotic parasites such as *Giardia*, *Cryptosporidium *and *Echinococcus *were not investigated in this phase of the study.

## Discussion

The findings of the present study show a widespread distribution of canine faeces throughout the city of Naples. However, there was a strong spatial gradient with 48% of the total variability accounted by between neighbourhood variability. The highest significant contamination was found in high density residential neighbourhoods in the south-western part of the city. This can be explained by the fact that these neighbourhoods generally have a large number of dogs (both owned and stray dogs) that contaminate the environment with their faeces. The positive association between the number of canine faeces and the human density is important to draw the attention of community administratives to the regular cleaning of the streets in areas of highest human population.

A limitation of the present study is that we included in the analysis simply human density and gender. Evaluation of more demographics than these latter, as well as socioeconomic factors, for association with faecal contamination may have uncovered new insights. In addition, other findings that would have been of interest in Naples include evaluation of the association of specific environments (e.g. pavement, vegetation, dirt, etc.) with the presence of faeces and possibly risk behaviours of humans (children playing in dirty areas, people walking barefoot, etc.). Unfortunately, we did not have such data at our disposal.

The parasitological results showed the presence of eggs of *T. canis*, *T. leonina*, *A. caninum *and *T. vulpis*, as well as oocysts of *I. canis*, in the faecal samples examined. Other studies which were conducted in various locations around the world have produced results similar to ours regarding the presence of parasitic elements in urban areas [5,6]. In Italy, the presence of these parasites has been recently reported in a study of canine faecal contamination in five urban areas (Camerino, Matelica, Messina, Sassari, and Teramo) [11], and in a study conducted in the city of Bari [12].

Although it is not possible to compare the present study directly with other surveys because of the different sampling and detection methods used, however the percentage of samples positive to parasites reported in the present study (16.9%) is lower than the values reported in similar studies in Argentina (40–70%) [5,8] as well as in the five Italian urban areas aforementioned (up to 47%) [11] but higher than those reported in public green areas from Bari [12].

Most other studies have involved collection of samples *per rectum *or from dogs at necropsy; however, our aim was to study canine faecal contamination as well as parasitic risk.

It is important to note that we found parasitic elements which are widely known as potential agents of zoonosis, i.e. *T. canis *and *A. caninum*.

With respect to *T. canis*, that human infection occurs in Italy is revealed by serological findings; antibodies prevalence ranged between 1.1% and 3.98% in healthy people from northern and central Italy [13-16], whereas it was 8.1% in symptomatic individuals from the urban area of Ancona (central Italy) [16].

Concerning *A. caninum*, a noteworthy outbreak of cutaneous *larva migrans *involving 6 people in Naples has been recently described by Galanti et al. [17]; the infection was contracted via contact with material used for floral arrangements which was probably contaminated with dog faeces.

In addition, the presence of *T. vulpis *eggs in 10% of the faecal samples examined should not be neglected given the zoonotic role of this whipworm [18].

## Conclusion

In conclusion, the results of the present study, conducted using GIS both for planning and sampling and for evaluation and presentation of findings, showed the presence and intensity of canine faecal contamination in the city of Naples, and the occurrence of canine parasitic elements, some of which are potential agents of zoonosis. The presence of canine faeces and their contamination with parasitic elements are neither unusual nor unexpected findings in urban areas worldwide (a review of the Italian situation have been recently reported by Poglayen and Marchesi [19]). However, our results presented per sub-area of the city are of considerable value to local public health and sanitation administratives to target prevention strategies. It is imperative that dog owners are informed about the potential risk posed by canine faeces in order to better prevent the transmission of zoonotic diseases.

## Methods

### Study area and Geographical Information Systems

The study was conducted between February and May 2005 in Naples (40°51'N; 14°16'E), a city located in the Campania region of southern Italy, covering an area of 117.14 km^2^, with 1,004,500 inhabitants (data from the Italian Statistics Institute – ISTAT, 2001) and a dog population of about 30,000. The city is divided into 21 neighbourhoods (minimum neighbourhood area = 1.01 km^2^; maximum neighbourhood area = 11.45 km^2^). A GIS was constructed utilizing the geo-referenced digital aerial photographs and the cadastral maps (1:5,000) of the city of Naples (source: Cartographic Office of the Campania region). In order to uniformly evaluate the canine faecal contamination throughout the entire city, a grid representing sub-areas of 1 km × 700 m was overlaid on the city map within the GIS. This grid dimension was chosen to match the city cadastral maps. As a result, the territory of Naples was divided in 218 equal, rectangular sub-areas. In each sub-area we chose the straightest 1 km transect, and subsequently we digitalized it on-screen in the GIS.

The 218 sub-areas were divided in three subsets and assigned to three private veterinarians who were familiar with the study area. They screened their sub-areas and counted the canine faeces along the 1 km transects (indicated on the cadastral maps given to them) and collected the samples. The GIS software used for the present study was ArcGIS 8.3.

### Counting of canine faeces

Out of the total 218 sub-areas, we investigated 143 sub-areas (66%) because the other 75 sub-areas were inaccessible zones, for example, fast flowing traffic avenues, private property, railway track, etc. All the canine faeces counts were performed between 7.00 and 9.00 a.m. Counts were not performed on days of heavy rains.

### Sampling of canine faeces

In each sub-area, when possible, 3 canine faecal samples were collected (into polythene bags) along the 1 km transect (one at the beginning, one at the middle and one at the end of the transect) for coprological examinations. The number of samples was the same for lightly and heavily contaminated sub-areas.

The total number of samples collected was 415. We used a handheld Geographic Positioning System (GPS GARMIN 12XL, distributed by Garmin International Inc., 1200 East 151st Street, Olathe, KS, USA) which allowed to identify the precise geographical location of the canine faeces collected with an accuracy of <40 m.

### Laboratory coprological examination

Each of the 415 canine faecal samples collected was individually examined.

Microscopic coprological examination employed a novel faecal egg count technique, the FLOTAC technique [20] with an analytic sensitivity of 1 egg/oocyst/larva per gram (EPG/OPG/LPG) of faeces. We used a sodium nitrate solution (density = 1.200) to detect and count *T. vulpis *eggs, and a sodium chloride solution (density = 1.200) to detect and count other parasitic elements. It should be noted that the FLOTAC technique and the aforementioned two flotation solutions were selected based on their efficacy in floating the parasitic elements found in dog faeces as determined by us in an unpublished preliminary study (data not shown).

### Statistical analysis

a) Analysis of the spatial variability of the number of canine faeces and of the association with human population density (dependent variable: faeces counts; statistical model: negative binomial and Gaussian random effects regression).

Faeces count was modelled as over-dispersed Poisson, using a random effects negative binomial model [21,22]. Using this model the total variability was decomposed in between and within neighbourhoods. In details, the dependent variable was faeces count taken from a regular grid on the area under investigation. A substantial extra-Poisson variability of faeces count was expected due to non-homogeneous canine population density [23].

The same negative binomial regression model was used to evaluate the association between the number of canine faeces in the neighbourhoods (dependent variable) and the following independent variables: human population density (total number of inhabitants/km^2^), male and female population density in the neighbourhoods. Those latter were the only demographic data at our disposal. No model selection strategy was applied. We fitted separately each covariate at turn. Their contribution was assessed by likelihood ratio test. To check for assumptions this analysis was replicated fitting a Gaussian random effects model on log-transformed faeces counts [24].

b) Analysis of the association between faeces positivity and number of faeces in the neighbourhood (dependent variable: positivity to parasitic elements, yes/no; statistical model: logistic regression)

A further analysis was undertaken to study determinants of positivity to parasitic elements (yes/no, binary dependent variable). A logistic regression model was fitted to evaluate the association between positivity to parasitic elements and number of canine faeces in the neighbourhoods (as a continuous covariate).

All the analyses were done with SPSS 13 software for Windows and STATA 9.

### Data mapping

In order to display the results, a choroplethic map and a point map were drawn within the GIS [9]. In the choroplethic map, a continuous grey scale represents the total number of canine faeces counted in the 1 km transect of the sub-areas. We divided the scale into 4 grey intensities. The white sub-areas represent the absence of canine faeces, the hatched ones represent sub-areas for which there were no data. The point map shows the geo-referenced points in which canine faeces were collected; faeces negative for parasitic elements were represented in white; those positive for zoonotic parasitic elements in black, and those positive for non-zoonotic parasitic elements in grey.

## Authors' contributions

LR analyzed the results and drafted the manuscript. AB and DC carried out the statistical analyses. SC and VV contributed to acquire the samples and to carry out laboratory analyses. VM performed the maps by GIS. GC performed the study design and sampling, helped to draft the manuscript and revised it. All authors read and approved the final manuscript.

## Figures and Tables

**Table 1:** Parasite species found in the canine faecal samples, Naples, Italy, February–May 2005.

**Parasite species**	**Sub-areas (No. examined = 143)**			**Faecal samples (No. examined = 415)**			**EPG/OPG (percentiles)**		
			
	**No. positive**	**%**	**95% CI**	**No. positive**	**%**	**95% CI**	25^th^	50^th^	75^th^
			
*Trichuris vulpis*	37	25.9	19.1, 34.0	42	10.1	7.5, 13.5	2.0	11.0	71.0
*Ancylostoma caninum*	8	5.6	2.6, 11.1	10	2.4	1.2, 4.5	3.5	7.0	14.0
*Toxascaris leonina*	5	3.5	1.3, 8.4	6	1.4	0.6, 3.3	3.5	35.0	424.0
*Toxocara canis*	2	1.4	0.2, 5.5	3	0.7	0.2, 2.3	14.0	16.0	21.0
		
*Isospora canis*	15	10.5	6.2, 17.0	17	4.1	2.5, 6.6	2.0	4.0	7.0
